# Study‐design in pandemics: From surveillance and performance‐evaluation to licensing and pharmacovigilance

**DOI:** 10.1002/pst.2217

**Published:** 2022-07-12

**Authors:** Sheila M. Bird

**Affiliations:** ^1^ MRC Biostatistics Unit Cambridge UK; ^2^ College of Medicine and Veterinary Medicine University of Edinburgh Edinburgh UK

**Keywords:** licensing, pandemic, performance‐evaluation, pharmacovigilance, surveillance

## Abstract

Andy Grieve, the first pharmaceutical statistician to be President of the Royal Statistical Society, practiced in the regulated world of drug development. With reduction in drug development costs as his motivation, Grieve advanced Bayesian methods for developing predictive methods for efficacy and toxicity ‐ to be used as early as possible in the drug development process; and his presidential address exhorted statisticians to weigh‐in wherever data are used to make decisions. Diagnostic tests for infectious diseases are less regulated than drugs and vaccines unless the blood supply is at risk. Unlike in the HIV and HCV pandemics of the late 20th century, even well‐designed surveys linked to a volunteered biological sample (to be tested for SARS‐CoV‐2 antigen or antibodies) have had modest or low consent rates. Record‐linkage, statistical design and reporting standards have seen triumph and tragedy. Among the triumphs are: Liverpool's insistence on dual testing (lateral flow device; polymerase chain reaction [PCR]) of some 6000 asymptomatic citizens who attended for SARS‐CoV‐2‐screening; two tricky randomized controlled public‐policy trials on daily contact testing for close contacts of index cases of SARS‐CoV‐2 infection versus self‐isolation (with or without initial PCR); and among already‐consented participants in surveillance, over 80% secondary consent for linkage to their health records, including the Immunization Management Service. Before the next pandemic we need to entrench better regulation of diagnostic tests, better informed consent (not via weblinks), better feedback to participants, and transparency about basic safety data.

## 1 INTRODUCTION: STATISTICAL REPORTING STANDARDS, STUDY‐DESIGN, AND RECORD‐LINKAGE

As a biostatistician, statistical reporting standards (particularly in medical journals^1^, ^2^) and designing studies to ensure high volunteer‐rates, including by feedback of results to participants,^3^, ^4^, ^5^ have been key concerns. Later came discoveries, about public health, epidemics and pharmacovigilance, through no‐names record‐linkage studies which, although unconsented, were approved because in the public interest.^6^, ^7^, ^8^, ^9^, ^10^

A patchwork of reports by Royal Statistical Society (RSS) Working Parties in the past 30 years crafts together the above themes: Official Statistics – Counting with Confidence^11^ (which led to UK Statistics Act and UK Statistics Authority); Statistics and Statisticians in the Pharmaceutical Industry^12^ (which led to the appointment of professional statisticians by UK's drug regulator; and a decade later by European Medicines Agency); Performance Monitoring in the Public Services^13^ (which called for greater use of experimental methods in policy‐evaluation); Statistical Issues in First‐in‐Man Studies^14^ (which required study‐designs to specify that sufficient time elapsed between the administration of drug/placebo to successive volunteers so that never again would more than one healthy volunteer, far less six, have to be admitted to intensive care); and most recently Diagnostic Tests^15^ (which made recommendations on study‐design, regulation and transparency).

## 2 REFLECTION ON RSS'S FIRST PHARMACEUTICAL‐STATISTICIAN PRESIDENT

Andy Grieve's term of office as RSS President was 2003–2005. Timing was opportune: after the appointment in the mid‐1990s of pharmaceutical‐statistician, John Lewis, to lead a team of professional statisticians at UK's Medicines and Healthcare products Regulatory Agency (MHRA); after the set‐up in 1999 of the National Institute for Clinical Excellence (NICE); and coincident with advocacy by the RSS's Performance Monitoring Working Party—via workshops and parliamentary roadshows—of properly‐designed performance monitoring protocols, robust reporting standards including uncertainty and formal experiments for policy‐evaluation. Professor Grieve, his design credentials having been honed both in academia and by the rigor and efficiency required in the pharmaceutical industry,^16^, ^17^ was enormously supportive.

As a Medicines Commissioner for 5 years from 1991 (and former member of the RSS Working Party on Statistics and Statisticians in Drug Regulation), my practice after each meeting of the Medicines Commission was to write to the then‐head of MHRA to set out statistical issues which had been overlooked by his assessors. Unbeknown to me, Professor Michael Rawlins as chair of the Committee on the Safety of Medicines, was also advocating for the appointment of professional statisticians to MHRA. Two sources of continuous drops soon wore away the stone of resistance and MHRA made an excellent appointment in John Lewis. In 1999, NICE was set up. Under its inaugural 13‐year chairmanship by Professor Sir Michael Rawlins, NICE progressively established global standards (statistically, economically and on transparency) for the assessment and appraisal of cost‐effectiveness.^18^, ^19^ Notably, cost‐effectiveness models have to be operable by NICE‐assessors so that their coding and assumptions can be checked; and inputs changed to facilitate sensitivity analysis.^20^

As the first statistician appointed to the NICE Appraisal Committees, I gave an invited lecture (Trials and Tribulation: Judicial, Ethical and NICE) to the 2001 conference of Statisticians in the Pharmaceutical Industry. The conference was remarkable for my meeting (in the loo) Victoria Beckham with her young son – because England players were staying at the same hotel; and for my breakfasting with Andy Grieve and John Lewis. Full of enthusiasm, I explained that my talk would focus on the novelty and excitement that cost‐effectiveness at NICE represented for pharmaceutical statisticians: by designing‐in cost‐effectiveness considerations from outset of a pharmaceutical research programme. Andy and John looked at each other: who was going to burst my bubble?

They warned me that the talk would go down like a lead balloon (and it did!): that marketing teams rather than statistician‐teams were dealing with cost‐effectiveness at the end rather than at the start of pharmaceutical research programmes; and that statisticians in the pharmaceutical industry liked the assurance of familiar designs, not the risks that novel approaches posed. Twenty years later, the 2021 Armitage Lecturer at MRC Biostatistics Unit, Professor Gianluca Baio from University College London, who currently serves on the NICE Appraisal Committees, made a similar case in his oration on Statistical Modeling for Health Technology Assessment and the Analysis of the Value of Information.

Cost‐effectiveness and NICE were part of the RSS's outreach to journalists. Andy gave the all‐important first of the afternoon's engaging short talks, (10 minutes each and 10 minutes for questions, which journalists are not shy in posing). Topics ranged from randomized controlled trials (Phase I to Phase IV), efficacy versus effectiveness, the public's valuation of scores for the European Quality of Life on 5 Dimension (EQ5D), NICE's adoption of EQ5D to derive quality‐adjusted life‐years (QALYs), and assessment versus appraisal of cost‐effectiveness.^19^

Tim Holt had succeeded Andy Grieve as RSS President. In March 2006, I was alarmed by an item on Radio 4's TODAY programme: six healthy volunteers had been admitted to intensive care at Northwick Park Hospital during a first‐in‐man study of the monoclonal antibody TGN1412. Their admission was being described as “rare.”

Urgently, I phoned Nigel Hawkes, Health Editor at *The Times*, to explain that there had to be a serious error in the study's design for six to be admitted: one would be rare, six was unprecedented. Nigel ran copy for days raising questions about the TGN1412 study‐protocol that had been approved by the UK's regulator. The RSS established a Working Party to address Statistical Issues in First‐in‐Man Studies,^14^ which Professor Stephen Senn chaired. Several of the RSS Working Party's key design‐recommendations, including the need for an adequate time‐interval between the dosing of successive healthy volunteers, were shared with, and incorporated in, the final report on 30 November 2006 by the government‐appointed Expert Group on Phase One Clinical Trials. The RSS Working Party advised that sponsors, and MHRA assessors independently, should classify the finally proposed doses of an investigational medicinal product by level of a priori assessed risk (low, medium or high), having regard to the confidence in preclinical data. High a priori risk would rule out participation by healthy volunteers. And a precautionary approach to study design is appropriate for any experimental medicine which is “first in class” (and therefore high a priori risk level) or for which a priori assessed dose‐specific risk is high. See also Grieve^21^ on being a well‐calibrated Bayesian in clinical drug development.

## 3 DESIGN IN PANDEMICS: SURVEILLANCE AND PERFORMANCE MONITORING

Three blood‐borne pandemics of the late 20th century not only affected the blood supply but also altered the licensing of some diagnostic tests^15^; and sharpened biostatistical thinking, mine included. Antibodies to human immunodeficiency disease (HIV) are detectable in blood; as are antibodies to hepatitis C virus (HCV). However, the abnormal prion protein (PrP^SC^) which causes variant Creutzfeldt Jakob Disease (vCJD) is transmissible by blood transfusion^22^; but, even now, is not detectable in blood. Leucodepletion of the blood supply and barring recipients of blood or tissue from donation safeguard the UK's blood supply.

The RSS advocated unlinked anonymous HIV surveillance^.^ Anonymous HIV surveillance studies based on a) antenatal or neonatal blood samples (both of which reflect maternal antibodies) or b) blood samples taken for syphilis testing from persons attending genitourinary medicine clinics were a framework for without‐consent (other than opt‐out*) surveillance for HIV prevalence in at‐risk sub‐groups for whom only minimal demographical information (gender, age‐group, region, calendar‐period) would be associated with residual blood samples (taken originally for another purpose that had already been fulfilled) which would be tested for HIV (later also HCV) antibodies.^23^

Notices about unlinked anonymous testing (UAT) were posted in clinics where UAT was operational so that patients could opt‐out* if they so wished. Opt‐out rates were typically low (around 1%) and UAT surveillance was highly informative about HIV prevalence in strategic sub‐groups and greatly assisted AIDS/HIV projections.^24^ Bizarrely, however, it was years before the results of UAT were posted in the participant clinics so that patients might track, regionally or nationally, the information to which they contributed. Feedback was especially important, and the lack of it remiss, because UAT was usually able to estimate the proportion of patients who were HIV‐infected but had not been diagnosed – knowledge that could have encouraged patients to request a personal HIV test.

By contrast, the results of our Willing Anonymous Salivary HIV/HCV (WASH) studies in Scottish prisons were reported back, per prison, within 6 weeks: to prisoners and prison‐governor at the same time. Prisoners themselves linked their saliva sample (to be tested for HIV antibodies) to their no‐names self‐completion HIV‐risk questionnaire and did so by selecting an envelope from a bag of 50. Within the envelope was a pair of sealed labels. Under the seal was the same number (e.g., A666) on each label but neither the prisoner nor anyone else knew what the number was. The prisoner put one label on the salivette containing his/her saliva sample, the other on the risk‐factor questionnaire which was then folded into the envelope. On exiting the surveillance area (usually the prison's gymnasium), prisoners deposited their salivette in a big red bin (to go to HIV Immunology Laboratory), their enveloped risk‐questionnaire in a big blue bin (to go to MRC Biostatistics Unit) – thereby separating the two. The laboratory reported to the statistician‐team the unsealed number for samples which had tested positive for HIV antibodies. Prisoners were willing to take part because they appreciated the demonstrable anonymity that the WASH surveillance design afforded them^3^, ^4^, ^5^ and knew that the results for their prison would be reported‐back to them by us within 6 weeks. Volunteer‐rates were high: generally over 80%.^25^, ^26^, ^27^

Our WASH surveillance studies in 1996, at prisons in Aberdeen and Lowmoss, got us into trouble.^27^ For the first time, we asked prisoners who had been inside for the past 4‐weeks how often they had injected inside prison in the past 4‐weeks; and how often in the past 4‐weeks they had used sterilization tablets to clean needles and works. Inside‐injectors averaged 6 times in 4‐week (*SD* also 6). Random mandatory urinary drugs testing (rMDT) of prisoners had not yet begun in Scotland. We had warned civil servants and Ministers that, since cannabis stays in the urine for 2–3 weeks, heroin for 2–3 days, rMDT risked setting up a new market for inside‐use of heroin since punishment for testing positive for cannabis cost 2 weeks loss of remission, for heroin 3 weeks. Moreover, since rMDT did not operate at weekends, savvy injectors would be liable to test positive for heroin on as few as 9 days or as many as 18 days out of 28. Hence, rMDT under‐estimated inside‐use of heroin by a factor of 1.5 to 3. Our punishment? Barred from doing studies in Scottish prisons for 3 years…

Which partly explains why our attention turned to record‐linkage studies to quantify the risk of overdose‐death soon after release from Scottish prison custody. Using record‐linkage techniques, Seaman et al. had shown that, for Edinburgh's HIV‐infected male injectors, the risk of overdose death was eight times higher in the first fortnight after release from Edinburgh Prison than at comparable other times at liberty.^6^ Understandably, the question was raised: does the same apply to injectors who, for the most part, are not HIV‐infected? Hutchinson and Bird confirmed that, in 1996–99, the risk of male drugs‐related death was seven times greater in the first fortnight after release from Scottish prison custody than per fortnight in the next 10 weeks (95% CI: 3–16); and estimated that, at the end of the 20th century, 1 in 200 adult male injectors was dead from overdose with the first fortnight following release from Scottish prison custody.^7^ Opioid substitution therapy as healthcare standard for the Scottish Prison Service from 2003 and Scotland's National Naloxone Programme (NNP) from 2011 have made impressive differences – as revealed by before/after studies.^28^, ^29^ The NNP‐related 30% reduction (at least) in the percentage of opioid‐related deaths with a 28‐day antecedent of prison‐release, see Table 1 was apparent sooner than by the contemporaneous N‐ALIVE trial of naloxone‐on‐release.^30^

**TABLE 1:** Five‐years before (2006–2010) and the first (2011–2013) & second (2014–2016) 3‐years after the set‐up of Scotland's National Naloxone Programme: primary, secondary, and tertiary outcomes

Scotland's National Naloxone Programme: Outcomes				
Calendar period (duration); naloxone kits issued, including to prisons	Number of opioid‐related deaths, ORDs	Primary: ORDs within 4‐weeks of prison‐release (as % of ORDs)	Tertiary: ORDs within 4‐weeks of hospital‐discharge (as % of ORDs)	Secondary: ORDs within 4‐weeks of prison‐release and/or hospital‐discharge
2006–2010 (5 years)	1970	193	191	374
		9.8% (8.5%–11.1%)	9.7% (8.4%–11.0%)	19% (17.2%–20.7%)
2011–2013 (1st 3 yrs); 12,000	1212	76	111	181
		6.3% (4.9%–7.6%)	9.2% (7.5%–10.8%)	15% (12.9%–16.9%)
2014–2016 (2nd 3 yrs); 24,000	1592	60	151	204
		3.8% (2.8%–4.7%)	9.5% (7.7%–11.4%)	13% (11.2%–14.5%)
2011–2016 (6 years); 36,000	2804	136	262	385
		4.9% (4.1%–5.6%)	9.3% (8.2%–10.4%)	14% (12.4%–15.0%)

Properly informed consent and feedback to participants on the results of surveillance matter. During the COVID‐19 pandemic, participation‐rates dropped from around 40% to 15% in well‐designed, representatively‐sampled national surveillance studies, such as household‐sampling by the Office for National Statistics (ONS) Community Infection Survey (CIS, with re‐imbursement of participants) or England's REal‐time Assessment of Community Transmission‐1 (REACT‐1) series of community surveys for SARS‐CoV‐2 infection, based on RT‐PCR results from self‐administered throat and nose swabs from over 100,000 participants and linked self‐completion questionnaire. Each round of REACT‐1 invited a random cross‐section of the population of England aged 5+ years. The sampling frame for REACT‐1 was the National Health Service (NHS) list of patients registered with a general practitioner in England, which is held by NHS Digital.

REACT‐1's excellent analyses were released as MedRxiv pre‐prints, typically within 2 weeks of completion of each round. Even so, participation rate in REACT‐1 reduced from about one‐third initially to 12% in round 15 when 859,184 persons were invited to participate, of whom 143,193 registered (16.7%) and 100,112 provided a swab with valid RT‐PCR result, see https://spiral.imperial.ac.uk/bitstream/10044/1/92501/2/REACT1_Round15_Final.pdf. For the current and previous round, REACT‐1 orients readers by reporting unweighted and re‐weighted swab‐positivity. Optional consent is high (around 80%) for linkage to the participant's NHS record which enables detailed, insightful analysis of vaccine effectiveness by vaccine‐type and number of doses.

What explains the low participation rate in REACT‐1? Feedback to participants is limited to their RT‐PCR result. Might those invited to take part appreciate being given an illustrated handout about the main results of the previous round (akin to REACT‐1's Abstract) and a link to where corresponding highlights from their round can be accessed? Second, REACT‐1's letter of invitation, which I received in March 2021, was signed by (Lord) Bethell, Parliamentary Under Secretary of State at Department of Health and Social Care (DHSC), Professor the Lord Darzi FRS as Co‐Director of the Institute of Global Health Innovation at Imperial College London and Kelly Beaver as Managing Director for Public Affairs at Ipsos MORI.

The signatories reflect general eminence and influence rather than specific subject matter expertise. Thirdly, before and during the COVID‐19 pandemic, over‐long and poorly‐designed questionnaires have proliferated as a low‐cost means of assessing the public's understanding of, and non‐compliance with, public health and other guidance. During COVID‐19, response‐rates to such questionnaires (often below 10%) have had to be extracted from reluctant contractors. The public's tolerance for low‐quality “research” is rightly low. But the high‐quality of other online questionnaires, such as REACT‐1's, cannot be discerned until the citizen has signed‐up, a risk that many may be unwilling to take these days. Do we need kite‐marking of online questionnaires to alert the public to high‐quality surveys?

Fourth, properly informed consent should not require the citizen to trawl through a series of web‐links to discover critical details. Despite assurances about research confidentiality, legal powers apparently obliged REACT‐1 to disclose personal identifying information to NHS Test and Trace about research participants whose altruistic RT‐PCR test was negative. My complaint to the Health Research Authority, that REACT‐1's consent at round 10 had not been sufficiently explicit about the transfer of personal identifying information to NHS Test & Trace, was upheld. REACT‐1, of course, made the necessary changes and DHSC ensured the deletion of data about me that REACT‐1 had transferred to NHS Test & Trace.

There is, however, a broader issue: research ethics committees should ensure that properly informed consent is upfront so that critical details are not found only via a mist of weblinks. The same caveat about a mist of weblinks, especially if without effective version control, should apply when MHRA approves Information for Users of medicines or devices, see later.^31^

## 4 DESIGN IN PANDEMICS: PLATFORM TRIALS OF RE‐PURPOSED TREATMENTS, COVID‐19 VACCINE TRIALS, AND PUBLIC‐POLICY TRIALS

Three key platform trials of re‐purposed drugs began in early 2020 and have discovered effective treatments in COVID‐19. The three are as follows:

1. PRINCIPLE (higher risk patients in primary care trial). www.principletrial.org.
2. RECOVERY (in hospital trial) https://www.recoverytrial.net/ For further information please Email: recoverytrial@ndph.ox.ac.uk.
3. REMAP‐CAP (critically ill patient trial) https://www.remapcap.org/ For further information; Please Email: ukremap-cap@icnarc.org. (REMAP‐CAP stands for Randomized, Embedded, Multi‐factorial, Adaptive Platform Trial for Community‐Acquired Pneumonia).

As a large simple platform trial, RECOVERY is able to compare multiple treatments at the same time using a single protocol. This type of trial allows new treatments to be added, and treatments that are ineffective to be dropped, throughout the course of the trial, all the while respecting the principle of concurrent controls in the trial's statistical analysis plan, see https://www.recoverytrial.net/files/recovery-sap-v3-2-2021-12-17.pdf. RECOVERY, the largest of the above three platform trials, has recruited over 45,000 patients (nearest 1000). See https://www.isrctn.com/ISRCTN50189673.

“What does the RECOVERY study involve?If a patient decides to join, they will be asked to sign the consent form (for children, their parent/guardian will sign the consent form). Next, brief details identifying them and answering a few questions about their health and medical conditions will be entered into a computer. The computer will then allocate them at random (like rolling a dice) to one of the possible treatment options. Neither patients nor their doctors can choose which of these options will be allocated. In all cases, treatment will include the usual standard of care for the hospital. Please see the trial website https://www.recoverytrial.net/ for details of the current study treatments.”

From a standing‐start in January 2020, a remarkable compendium of COVID‐19 vaccines has been developed: either virus‐vectored (such as Oxford/AstraZeneca, OAZ^32^) or messenger RNA (mRNA, such as PfizerBioNTech, PBN^33^). First‐in‐man studies, dose‐scheduling studies and formal trials to quantify the efficacy of these vaccines against SARS‐CoV‐2 infection are discussed in detail elsewhere in this special issue. Hence, I make only three remarks: on randomization and statistical reporting standards.

### 4.1 Permuted randomization, fixed block‐length

Several Phase III COVID‐19 vaccine trials adopted permuted randomization with fixed block‐length, typically of length four. Examples are a Russian trial's 3:1 randomization (active: placebo)^34^ and a Chinese trial's equal randomization.^35^ Fixed block length means that the maximum imbalance can be summed across randomization‐strata (e.g., centres or age‐group within centre) and compared with the observed imbalance. Judging by published papers,^34^, ^35^ statistician‐referees may have forgotten to check for, or inquire about, tell‐tale signs that the reported method of randomization had delivered an aberrant outcome.

### 4.2 Tilted randomization, so that one‐quarter (say) receive their second dose on the original schedule

UK's MHRA licensed PBN in early December 2020 (with dose‐schedule 1/22 days, as in randomized controlled trial [RCT]) and OAZ in late December 2020 (with 2nd dose at 4–12 weeks after the first).

On December 31, 2020, UK's four Chief Medical Officers took a decision in the public interest that the inter‐dose interval would be maximized at 12 weeks for both vaccine‐types in order that all citizens in the four highest COVID‐19 risk‐group (health and social care workers, vulnerable patients and citizens aged 70+ years) should receive their first COVID‐19 vaccine‐dose by mid‐February 2021.^36^ Randomization to inter‐dose interval had apparently been considered: but was not adopted.

In early January 2021, I wrote to Ministers and England's Chief Medical Officer (CMO) to advocate tilted randomization to enable one‐quarter (say) of those receiving the world's first ever mRNA vaccine to receive their second dose according the RCT's 1/22 day schedule because the cited precedent (namely: longer dose‐interval, greater benefit) strictly applied to virus‐vectored vaccines only.^37^, ^38^

The case for randomization was accepted by the Deputy‐CMO and a National Institute for Health Research‐call was issued: for RCT powerful enough to identify a 20 percentage‐point difference in vaccine‐effectiveness by dose‐schedule, in the short or longer‐term. Timely submissions were received but funding for the call was suspended in February 2021 without the result of the competition ever being announced.

England (unlike Scotland) had been remarkably coy about disclosing, by vaccine‐type and epidemiological‐week jointly, the number of its citizens who had been vaccinated. As late as autumn 2021, modeling teams who reported to the COVID‐19 Scientific Advisory Group in Emergences (SAGE) made different assumptions about vaccine‐effectiveness by vaccine‐type (and also by elapsed time since second dose) against infection; and against hospitalization or COVID‐mention death conditional on infection.^39^ No record‐linkage analysis has been published on England's “Serendipity Cohort” of 650,000 citizens aged 80+ years who received their first PBN dose before January 4, 2021, half of whom (non‐randomized) received their second dose by January 25, 2021 (most by 10 January).^36^

### 4.3 Statistical reporting standards in pharmacovigilance

In April 2021 coyness again prevailed when MHRA announced that its pharmacovigilance had confirmed that the incidence of a serious adverse event, thrombosis with thrombocytopenia syndrome (TTS), in association with the OAZ COVID‐19 vaccine, was age‐related. Missing from MHRA's briefing, for each age‐group, were the following: numbers of OAZ first‐doses administered by DATE‐1, numbers of TTS cases reported by DATE‐2 who had received their OAZ first‐dose by DATE‐1, and numbers of TTS‐fatalities. Only modeled estimates from logistic regression were reported: no basic data, see https://assets.publishing.service.gov.uk/government/uploads/system/uploads/attachment_data/file/976877/CovidStats_07-04-21-final.pdf.

Within a week, actual counts were wisely leaked.^40^ The initial weakly‐evidenced decision—to offer the OAZ vaccine only to those aged 30+ years—turned out to be insufficiently precautionary as those aged 30–39 years were excluded soon thereafter. Concealment of basic data is an error of commission which denies proper independent scrutiny of how pharmacovigilance is delivered.

### 4.4 Public‐policy trials

The UK's failure in early 2021 to adopt tilted randomization for vaccine‐policy evaluation was disappointing. However, by mid‐2021, the UK could celebrate two major formal experiments evaluating public health policies on the deployment of daily testing of contacts (via antigen lateral flows devices [LFDs]) as alternative to self‐isolation (with or without PCR‐testing) after being notified as a close contact of a SARS‐CoV‐2 infected index case.

School‐based COVID‐19 contacts who are required to self‐isolate at home miss school days. English secondary schools and colleges, which already had in place weekly school‐based asymptomatic testing for SARS‐CoV‐2 infection using DHSC/INNOVA LFDs, were invited to take part in an open‐label, cluster‐randomized trial of daily testing of school‐based COVID‐19 contacts (with 24‐hour release if LFD‐negative), as alternative to self‐isolation at home, to assess whether the LFD‐intervention resulted in similar control of transmission (non‐inferiority margin: <50% relative increase) while allowing more school attendance.^41^ In all, 201 schools were randomly assigned during April 19, 2021 to May 10, 2021 (99 to control group, 102 intervention schools) in the 10‐week study, which ended on June 27, 2021. Most randomized schools participated actively (162/201). Importantly, the trial's access to national data (for example: results of routine SARS‐CoV‐2 PCR tests done outside the study in staff and students) enabled most non‐participating schools to be included in the analysis of co‐primary outcomes. In practice, only 42% (2432/5763) of the intervention group contacts participated in daily contact testing. Analyzed according to policy‐intention, the adjusted rate ratio (intervention: control) for symptomatic PCR‐confirmed infection was 0.96 (95% CI: 0.75–1.22); and for staff/student COVID‐related absences was 0.80 (95% CI: 0.54–1.19).

The triallists' 50% non‐inferiority margin was met but statistically significant reduction in COVID‐related absences was not demonstrated. This school‐based cluster‐randomized trial used the Orient Gene LFD for daily contact testing. Dedicated PCR testing was done in consenting contacts in both randomized groups on day 2 and day 7 of their testing or isolation period which enabled performance of Orient Gene LFD to be assessed via dual‐testing.^41^

In July 2020, the RSS COVID‐19 Taskforce had made recommendations on efficient use of statistical methods to glean intelligence from NHS Test, Trace and Isolate, including by the deployment of PCR‐testing on two random days (early and late) during self‐isolation, see https://rss.org.uk/RSS/media/File-library/Policy/RSS-COVID-19-Task-Force-Statement-on-TTI-final.pdf?ext=.pdf.

The STOP COVID RCT, see Box 1: About the study, was designed to determine the risk of onward transmission of SARS‐CoV‐2 infection (that is: to tertiary cases) from consented, randomized asymptomatic adult contacts of confirmed COVID‐19 cases. Randomization was to (a) serial self‐administered DHSC/INNOVA LFD plus two PCR tests, with 24‐hour release based on negative LFD (DCT intervention group, DCT) or (b) 10 days of isolation with one initial PCR (isolating with PCR group, PCR).

BOX 1 **About the STOP COVID trial**

**About the study:** Around 1 in 3 people who have COVID‐19 have no symptoms and could be spreading it without knowing. People who have been in contact with someone who has tested positive for COVID‐19 are more likely to be infected than those who have not.

A statement in March 2021 from the Scientific Advisory Group for Emergencies (SAGE) suggests that regularly testing contacts of confirmed cases is an effective way to reduce transmission of COVID‐19 and could potentially reduce the need for self‐isolation by contacts of positive cases.

The study will help Public Health England (PHE) and NHS Test and Trace understand:

- How useful daily contact testing is at finding new positive cases of COVID‐19.
- Whether it has an impact on transmission of the virus.
- How we could improve this service if we offered it to everyone in England.

The study is led by PHE and funded by the Department of Health and Social Care (DHSC). Its purpose is to find out if daily testing can replace the need for self‐isolating for people without symptoms if their test result is negative.

**How the study works:** Currently, everyone who's been notified by NHS Test and Trace that they have been in contact with someone who's tested positive for COVID‐19 in England must self‐isolate for 10 full days.

NHS Test and Trace will invite people who have been traced as a contact to take part in the study, providing they do not have symptoms.

The study will compare 2 approaches to routine testing of contacts:

- Participants in the ‘self‐isolation’ group will be given 1 PCR test. They must self‐isolate as normal for the full 10‐day self‐isolation period even if their test result is negative.
- Participants in the ‘daily testing’ group will be given 7 rapid lateral flow tests to test daily. They will be given 24‐h release from self‐isolation if the test is negative. They will also receive 2 PCR tests.

Participants will be placed into study groups at random.

Only participants in the daily testing group who continue to test negative and do not have symptoms are excused from the legal duty to self‐isolate each day. Participants in the daily testing group will have a legal duty to tell their employer that they are taking part in the study, and if they stop taking part for any reason.

As an employer, you can ask employees who are taking part not to come into the workplace if you choose.

**Who can take part:** People who are traced as contacts can take part if they:

- Do not have COVID‐19 symptoms;
- Live in England;
- Are not in full‐time education;
- Are aged 18 and over;
- Are not under the quarantine rules for arriving in England.

**What participants in the daily testing group need to do during the study:** If participants develop symptoms or test positive during the study, they must self‐isolate immediately and wait for the results of their follow‐up PCR test.

As contacts of positive cases, participants in the daily testing group have been instructed to follow additional safety measures while taking part. They'll be asked to reduce close contact with others as far as possible by only making essential trips outside the home for:

- Work or volunteering;
- Education;
- Buying food (if no one else can do it for them);
- Exercise in an outdoor space;
- A medical or personal emergency.

They'll be asked to avoid:

- Being in small, poorly ventilated public places for more than 15 min.
- Visiting others indoors.
- Using public transport, unless for essential trips.
- Visiting people who are clinically extremely vulnerable, in care homes or hospitals.

They'll be asked to further reduce risk of infection to others by:

- Opening windows wherever they can;
- Avoiding shouting, singing or talking loudly, particularly when indoors.

Randomization was household‐based for adult contacts living in the same household ‐ with the household's assignment determined by the allocation of the first adult contact in the household who both registered online for STOP COVID RCT and formally consented. In practice, “randomization” was alternation based on participants' haphazard online‐registration‐time (1–30 s; 31–60 s) with the assignment activated at the time when the participant pressed “consent”. Hence, descriptive statistics should include the distribution for first‐in‐household's registration‐second; and also the registration‐second distribution for the subset who formally consented.

Notification of assignment and issue of STOP COVID test‐kits was instigated twice‐daily (at 12‐hour intervals). Hence, the decision to participate by some later‐consented individuals may have been informed by prior knowledge of the household's randomized assignment. Such prior knowledge would favor DCT. Hence, sensitivity analysis is required, which fits an interaction between first versus later‐consented household members and randomized assignment of the first‐consented.

Within the DCT‐group, the performance of LFDs could be assessed at initial PCR or off‐study PCR (that is: having remained asymptomatic and LFD‐negative) or intermediate PCR (on account of DCT‐participant having tested LFD‐positive or developed symptoms). The proportion of participants who tested PCR‐positive (including on national database) and the proportion of their close contacts who were diagnosed with COVID‐19 (tertiary cases) were key outcomes with analysis by policy‐intention but also restricted to the first‐randomized adult contact per household. Publication of the results from this major, but tricky RCT which recruited around 50,000 adult close contacts is eagerly awaited.^42^

Both policy‐trials, each difficult to deliver, are a major break‐through in formal randomized evaluations of public policy^13^: and all the more impressive for having been designed and delivered during a pandemic.

## 5 DESIGN IN PANDEMICS: DIAGNOSTIC TESTS, LICENSING, AND POST‐MARKET VIGILANCE

The Royal Statistical Society's COVID‐19 Taskforce made a series of interventions concerning design issues in the evaluation of diagnostic tests, whether for SARS‐CoV‐2 infection or “infectiousness”; or for SARS‐CoV‐2 antibodies as a marker of very recent (IgM) or past (IgG or total) infection. See https://rss.org.uk/news-publication/news-publications/2020/general-news/rss-launches-new-covid-19-task-force/.

For example, Spiegelhalter wrote to MHRA with statistical advice on the specification of Target Product Profiles (and associated study size) to ensure that the lower 95% confidence limit (for sensitivity or specificity) excluded MHRA's chosen lower limit.

Next, on the eve of evidence on testing that Bird and Deeks were due to give to the Lords Science and Technology Committee Inquiry into the Science of COVID‐19 on June 9 2020, colleagues at Public Health England (PHE) provided frank answers to questions that we had raised, including as Science Media Centre posts, about the absence of an even playing field in how different antibody tests had been evaluated. Subsequently, PHE published an explanatory paper, warts and all, which clarified why the evaluation of four rival tests had evolved differently and the steps taken to ensure greater comparability between future evaluations. A key difficulty that PHE had faced was access to a bank of patients' biological samples, that could be randomly sampled from for use in more than one evaluation.

In January 2021, see above, questions were raised about the absence of a sufficient randomized control group (say, one‐quarter of those being immunized with PBN vaccine) when the UK's Chief Medical Officers decided to extend the inter‐dose interval from the originally‐licensed 3 weeks to 12 weeks for the roll‐out in the UK of the world's first‐ever mRNA vaccine.

In April 2021, see above, questions were raised about the basic evidence‐base (subsequently leaked) that led to MHRA's decision to advise that the Oxford/AstraZeneca vaccine be not administered in persons under 30 years of age, rather than the more precautionary threshold of under 40 years which was adopted about a month later.

Pharmaceutical medicines and vaccines require a licence from MHRA before they are prescribed for UK patients. During the SARS‐CoV‐2 pandemic, diagnostic tests have not had to pass the hurdle of licensing. Instead, Emergency Use Authorization, largely based on self‐certification, permits a diagnostic test to be deployed. Hence, in June 2021, the Royal Statistical Society's Working Group on Diagnostic Tests, made 22 recommendations: 10 on study‐design matters, six on regulation matters and six on matters of transparency. See https://rss.org.uk/RSS/media/File-library/Policy/2021/RSS-Diagnostic-tests-report-FINAL.pdf.

On the credit side, see above, two major public‐policy randomized controlled trials have been conducted on whether daily contact testing by LFDs enables 24‐hourly‐release from isolation without undue impact on onward transmission of SARS‐CoV‐2 infection. Empirical results could then be compared with prior modeling studies.^43^, ^44^

Three major statistical causes celebres on diagnostic testing have also occurred during November 2020 to October 2021.

### 5.1 Moonshot

With logistical support from military personnel and by dint of academic leadership from the University of Liverpool, the aspiration of the “Moonshot” programme was that three‐quarters of the asymptomatic citizens of Liverpool would come forward for weekly LFD‐screening at assisted‐testing‐sites. During the initial 4 weeks, one‐quarter of Liverpool's citizens took up the offer of LFD‐testing at least once (mostly once‐only): not the naively aspirational three‐quarters!

The INNOVA LFD was to be deployed for asymptomatic testing of Liverpool's citizens for 4 weeks from November 6, 2020 but had had limited prior evaluation in persons who did not have symptoms of COVID‐19. Moreover, its use for asymptomatic testing was outside of the INNOVA test developers' self‐certification. Hence, the University of Liverpool academic team had insisted that dual LFD/PCR testing by around 6000 consented asymptomatic citizens who came forward for screening would be needed to establish the INNOVA test's sensitivity for detection of SARS‐CoV‐2 infection when testees were asymptomatic. Dramatically, the team discovered that, of 70 dual‐screened asymptomatic citizens who tested PCR‐positive for SARS‐CoV‐2 infection, only 28 were detected by the INNOVA LFD so that INNOVA LFD's sensitivity for asymptomatic screening was only 40% (95% CI: 28%–52%). Specificity was high, however.^45^

Subsequently, goal‐posts were changed several times to assert better sensitivity for “infectiousness”, defined indirectly by the cycle‐threshold value (CT‐value) on PCR‐testing being 25 or lower (about 67% sensitivity); and better still, about 90% sensitivity, when CT‐value was 18 or lower. Prior to the Delta variant, only about one in six PCR‐positive persons had CT‐value of 18 or lower.…

Moonshot was operational when around 1% of Liverpool's asymptomatic citizens had tested PCR‐positive. In early 2021, the UK government continued to promote weekly or twice‐weekly INNOVA‐testing by social care staff and for other key workers in the public or private sector. Modeling studies formed part of the policy‐rationale; but contractors failed to deliver decently‐designed empirical evaluations.

By contrast, a team from PHE offered close contacts of SARS‐CoV‐2 infected index cases the opportunity to opt for daily contact testing (using DHSC/INNOVA test) during their isolation‐period. Participants undertook PCR testing if they developed symptoms, tested LFD‐positive or reached the end of their period of self‐isolation after testing LFD‐negative throughout.^46^ This non‐randomized study gave confidence that PHE could deliver on the requirement for a suitably powered randomized public‐policy trial of daily contact testing (to enable 24‐hourly release but corroborated by initial PCR‐test and another when LFD‐positive or at the end of isolation if persistently LFD‐negative) versus a control group with initial PCR‐testing only and were to self‐isolate.

### 5.2 Return to secondary schools in England, March 2021

Prevalence of SARS‐CoV‐2 infection was high at the end of January 2021 when England suspended the need for PCR‐adjudication of LFD‐positive test results: no doubt because most PCR‐adjudications were then PCR‐positive.

On March 5, 2021, the RSS COVID‐19 Taskforce issued its statement forewarning (correctly) that, due to low SARS‐CoV‐2 prevalence among secondary pupils on their return to school, half of secondary pupils' school‐assisted LFD‐positive test‐results would be negative on PCR‐adjudication, see https://rss.org.uk/RSS/media/File-library/News/2021/RSS-statement-on-surveillance-in-schools-5-March-2021.pdf. Secondary pupils in England were being asked to take three school‐assisted LFDs during the two‐weeks of March 8–19, 2021 before undertaking voluntary twice‐weekly LFD test at home thereafter.

The RSS COVID‐19 Taskforce was shocked the discover that, even if parents sought PCR‐adjudication and their child was PCR‐negative, NHS Test and Trace would ignore the adjudication and insist that the child did not attend school and the family maintained self‐isolation for 10 days. See Bird for a day‐by‐day account of this statistical cause celebre.^47^ Finally, PCR‐adjudication of LFD‐positives was restored at the end of March 2021.

Next, on April 8, 2021, the UK government made 7‐packs of DHSC/INNOVA LFDs freely available for twice‐weekly home‐use by asymptomatic citizens. Two naïve expectations were that citizens would (a) comply with twice‐weekly home‐use and (b) routinely register LFD‐negative test results. The UK government had revised its communication strategy to emphasize the need for PCR‐adjudication of LFD‐positives: both because the proportion of adjudications that would be PCR‐positive depended on the prevalence of SARS‐CoV‐2 infection (which Ministers had learned the hard way in March 2021, if not before) and because genomic analysis, critical for the tracking of new variants, required that a swab had been submitted for PCR‐testing.

### 5.3 LFD‐positives as “canary” in the IMMENSA gold‐mine

In September 2021, PCR‐adjudication of LFD‐positives was the canary in the mine that caused the public, particularly in the South West region of England, to alert journalists to an untoward high‐rate of PCR‐negative adjudications: despite high prevalence of SARS‐CoV‐2 infection according to surveillance by ONS‐CIS and REACT‐1.

The South West region probably accounts for less than 10% of the population of England. The NHS Test and Trace monitoring statistics for England in September to mid‐October 2021, see Table 2, revealed that the national PCR‐negative rate in PCR‐adjudications of LFD‐positive test results, of which there were around 40,000 per week, had risen from 8% through 12% to more than 16%.

**TABLE 2:** Self‐report home‐test Lateral Flow Device positives subject to PCR‐adjudication

Week in 2021	Self‐report home‐test kit LFT positives	Matched to non‐void PCR (M)	PCR‐negatives (N)	PCR‐negative rate (N/M × 100)
26 August to 1 September	39,780	29,533 (74%)	2442	8.3%
2–8 September	41,170	30,597 (74%)	2975	9.7%
9–15 September	37,561	27,547 (73%)	3469	12.6%
16–22 September	49,203	38,187 (78%)	4388	11.5%
23–29 September	52,894	42,442 (80%)	6538	15.4%
30 Sept. to 6 October	54,251	44,568 (82%)	7873	17.7%
7–13 October	63,168	51,741 (82%)	7393	14.3%
14–20 October	71,118	57,394 (81%)	4670	8.1%
21–27 October	59,556	47,110 (79%)	3492	7.4%

*Note*: About three‐quarters of self‐report home‐test LFD positives can be matched to PCR‐adjudications. The table above, for England, covers the period before/after PCR‐testing was suspended at a laboratory in Wolverhampton. On the basis around 40,000 PCR‐confirmations per week and PCR‐negative rate of 8%, the standard error for comparing between 2 weeks would be roughly 0.2%. Even without the IMMENSA hiatus, the data appear to be noisy so that there may be other factors in play, not least of which could be changing prevalence of SARS‐CoV‐2; and type of LFD (not currently in the public domain). For example, during mid‐October to early‐November 2021, weighted prevalence in round 15 of REACT‐1 was 1.57% (1.48%, 1.66%) compared to 0.83% (0.76%, 0.89%) in September 2021 (round 14). But increased prevalence of SARS‐CoV‐2 is likely to mean decrease (not increase) in the proportion of PCR‐adjudications which are PCR‐negative.

#### 5.3.1 Using 8% for pre‐IMMENSA and initial rise to 12% for illustration

If regions which accounted for only 10% of these PCR‐adjudications (that is: 4000 per week) were “responsible” in some sense for the increase, then the PCR‐negative rate in the affected area would have to have risen dramatically, say by a factor D, such that:

$$0.08\times \left[0.10\times D+0.90\right]=0.12=>\left[0.12/0.08–0.90\right]\times 10=D=>0.6\times 10=D\;\mathrm{so}\;\text{that}\;D=6,$$

which would mean that, other things being equal, the PCR‐negative rate in the “responsible” areas had increased from 8% to nearly 50%.

{Even if “responsible” regions accounted for 30% of PCR‐adjudication (not 10%), then D = 0.8 × 10/3 = 2.67, which would have meant a still notable, and noticeable, increase (as presumably based on 12,000 PCR‐adjudications per week) from 8% to 21% in the PCR‐negative rate for the “responsible” laboratory.}

Little wonder that the public noticed! Especially as many of those who apparently tested PCR‐negative would have developed symptoms and some would have felt very unwell indeed.

We now know that the IMMENSA Laboratory in Wolverhampton was responsible for the vast majority of these incorrect PCR‐negative test‐results; and that the problem dated from when the laboratory was asked to undertake PCR‐testing for the NHS using a different PCR‐technology than IMMENSA had been accustomed to when charging international travelers for private PCR‐tests.

What the yellow canary has brought to high‐pitch attention is how initiation‐training, internal quality controls, external quality‐assurance and inter‐laboratory comparison of PCR‐test‐performance could all have failed so dramatically: especially when the IMMENSA laboratory was being initiated into a new PCR technology in a new context of operations.

The questions raised about performance monitoring in the public service go well beyond the IMMENSA Laboratory and leave NHS Test and Trace lacking in oversight. Potential legal liabilities seem to have delayed public accountability. Legal liability may rest with Her Majesty's Government (HMG) if private providers of diagnostic testing in pandemic times have indemnity against rare forms of performance failure: akin to vaccine developers' indemnity against rare serious adverse events.

## 6 DISCUSSION

During pandemics, modeling studies—albeit with uncertain behavioral inputs and subject to sensitivity analyses—can deliver a useful prior distribution for the likely effect‐size that policy‐evaluations need to target.

Delayed transparency defers public and professional scrutiny of decisions: policy‐decisions or safety‐determinations by regulators. We need a better regulated world for diagnostic tests, especially in respect of infectious diseases with pandemic potential.

The public needs better guidance on the high quality of some (but not other) surveillance studies linked to biological samples so that participation rates in excess of 60% become routine, not exceptional.

Better designed surveys—in the public interest, with fewer but well‐posed questions—together with the promise of prompt feedback to respondents may help to improve volunteer‐rates.

As Grieve^17^ remarked, since statistics is the science of uncertainty and variability, an understanding of both is crucial to ensure rational decision‐making. Whether by Ministers, parliamentarians, public or press.

## CONFLICT OF INTEREST

Sheila M. Bird serves on RSS COVID‐19 Taskforce, on UKHSA's Testing Initiatives Evaluation Board and on RSS Working Group on Diagnostic Tests. SMB holds GSK shares.

## Data Availability

Data sharing is not applicable to this article as no new data were created or analyzed in this study. No original data included and so exempt.
